# COVID-19 Vaccine Hesitancy in Australian Patients with Solid Organ Cancers

**DOI:** 10.3390/vaccines10091373

**Published:** 2022-08-23

**Authors:** Nathan Bain, Mike Nguyen, Lisa Grech, Daphne Day, Amelia McCartney, Kate Webber, Alastair Kwok, Sam Harris, Hieu Chau, Bryan Chan, Louise Nott, Nada Hamad, Annette Tognela, Craig Underhill, Bao Sheng Loe, Daniel Freeman, Eva Segelov, on behalf of the CANVACCS Investigators

**Affiliations:** 1 Department of Oncology, Monash Health, Clayton, VIC 3168, Australia; 2 Department of Medicine, School of Clinical Sciences, Monash University, Melbourne, VIC 3168, Australia; 3 Department of Medical Oncology, Bendigo Health, Bendigo, VIC 3550, Australia; 4 Department of Oncology, Latrobe Regional Hospital, Traralgon, VIC 3844, Australia; 5 Department of Oncology, Sunshine Coast Hospital and Health Service, Birtinya, QLD 4575, Australia; 6 School of Medicine, Griffith University, Birtinya, QLD 4575, Australia; 7 Icon Cancer Centre Hobart, Hobart, TAS 7000, Australia; 8 Department of Hematology, St Vincent’s Hospital Sydney, Darlinghurst, NSW 2010, Australia; 9 School of Clinical Medicine, Medicine & Health, University of New South Wales, Kensington, NSW 2052, Australia; 10 School of Medicine, University of Notre Dame Australia, Chippendale, NSW 2007, Australia; 11 Macarthur Cancer Therapy Centre, Campbelltown Hospital, Campbelltown, NSW 2560, Australia; 12 Border Medical Oncology Research Unit, Albury, NSW 2640, Australia; 13 Rural Medical School, University of New South Wales, Albury, NSW 2640, Australia; 14 The Psychometrics Centre, University of Cambridge, Cambridge CB2 1AG, UK; 15 Department of Psychiatry, University of Oxford, Oxford OX3 7JX, UK; 16 Oxford Health National Health Service Foundation Trust, Oxford OX3 7JX, UK

**Keywords:** COVID-19, vaccination, vaccine hesitancy, cancer

## Abstract

Background: Vaccination is the cornerstone of the global public health response to the COVID-19 pandemic. Excess morbidity and mortality of COVID-19 infection is seen in people with cancer. COVID-19 vaccine hesitancy has been observed in this medically vulnerable population, although associated attitudes and beliefs remain poorly understood. Methods: An online cross-sectional survey of people with solid organ cancers was conducted through nine health services across Australia. Demographics, cancer-related characteristics and vaccine uptake were collected. Perceptions and beliefs regarding COVID-19 vaccination were assessed using the Oxford COVID-19 Vaccine Hesitancy Scale, the Oxford COVID-19 Vaccine Confidence and Complacency Scale and the Disease Influenced Vaccine Acceptance Scale-6. Results: Between June and October 2021, 2691 people with solid organ cancers completed the survey. The median age was 62.5 years (*SD* = 11.8; range 19–95), 40.9% were male, 71.3% lived in metropolitan areas and 90.3% spoke English as their first language. The commonest cancer diagnoses were breast (36.6%), genitourinary (18.6%) and gastrointestinal (18.3%); 59.2% had localized disease and 56.0% were receiving anti-cancer therapy. Most participants (79.7%) had at least one COVID-19 vaccine dose. Vaccine uptake was higher in people who were older, male, metropolitan, spoke English as a first language and had a cancer diagnosis for more than six months. Vaccine hesitancy was higher in people who were younger, female, spoke English as a non-dominant language and lived in a regional location, and lower in people with genitourinary cancer. Vaccinated respondents were more concerned about being infected with COVID-19 and less concerned about vaccine safety and efficacy. Conclusions: People with cancer have concerns about acquiring COVID-19, which they balance against vaccine-related concerns about the potential impact on their disease progress and/or treatment. Detailed exploration of concerns in cancer patients provides valuable insights, both for discussions with individual patients and public health messaging for this vulnerable population.

## 1. Introduction

Globally, there have been over 500 million SARS-CoV-2 infections and 6 million deaths during the pandemic as of March 2022 [1]. People with cancer are particularly vulnerable due to immunosuppression from the underlying disease and treatments and comorbid medical conditions [2]. A recent systematic review and meta-analysis of 81 studies of 61,532 patients reported that people with cancer are at approximately two times greater risk of serious illness and death from COVID-19 compared to the general population [3].

Vaccination is a critical component of the public health response to the pandemic [1]. COVID-19 vaccines have demonstrated efficacy in reducing infection, serious illness and death, and evidence is growing for real-world effectiveness of community-wide vaccination programs [4,5,6,7]. Pivotal clinical trials excluded patients with cancer, yet they were prioritized in vaccination programs due to the higher risk of adverse outcomes [8,9,10]. There is emerging evidence regarding safety and efficacy of vaccines in this cohort [11,12,13].

Globally, numerous studies report differing rates of willingness to receive COVID-19 vaccines, including in people with underlying health conditions [14,15]. The decision to accept a vaccine is multifaceted with influences including the motivation for health protection, hesitancy, uncertainty about effects, ease of access and desire for individualized advice [16]. Vaccine hesitancy, defined as “a delay in acceptance or refusal of vaccination despite availability of vaccination services”, ref. [17] has long been recognized as an important issue impacting vaccine uptake and the control of vaccine-preventable diseases [18]. Indeed, vaccine hesitancy was declared one of the top threats to global health by the World Health Organization in 2019 [19]. Vaccine hesitancy is a complex phenomenon influenced by sociodemographic and attitudinal factors including age, gender, education, socioeconomic status, beliefs about vaccines and trust in institutions [20,21].

Amongst people with cancer, vaccine hesitancy is generally lower than in the general population [15,22,23], although the decision-making impact of a cancer diagnosis on vaccination needs further exploration. People with cancer have expressed a range of concerns involving the impact of vaccines on the patients’ underlying cancer and treatments, as well as the potential effect of anti-cancer treatments on vaccine efficacy [24,25,26,27]. Concern regarding vaccine-associated side effects and the potential to interfere with treatment schedules is also prominent [24,26,28,29,30]. However, these cancer-specific factors and their influence on decision making have not been comprehensively analyzed using validated assessment tools to assess vaccine hesitancy in the context of cancer.

To better understand vaccine hesitancy regarding COVID-19 vaccination in people with cancer, we conducted a large multicenter survey exploring how the cancer-specific context impacted vaccine attitudes and behavior. The use of three validated vaccine hesitancy scales allowed a detailed understanding of factors influencing vaccination attitudes and the decision-making process.

## 2. Materials and Methods

### 2.1. Participants and Study Procedures

An internet-based cross-sectional survey hosted on the secure data capture platform Qualtrics [31] was conducted from 30 June to 5 October 2021. Eligible participants were aged 18 or over, with a past or current diagnosis of a solid organ or hematological malignancy, diabetes, or multiple sclerosis [22]. In this publication, we report on data from participants with a past or current diagnosis of a solid organ malignancy. Participants were recruited using a convenience sampling approach through nine Australian health services across four states, encompassing both public and private services in metropolitan and regional locations. The invitation to participate was sent by short message service to all patients with oncology clinic appointments in the various health services scheduled within the next six months and contained a link to the survey. Patients were also invited by clinicians during consultations, and promotional materials were displayed at the health services. Tablet devices and paper surveys were available at some sites. No incentives or remuneration was provided for participation. After providing informed consent, participants completed eligibility questions and were directed to the survey. The survey was presented in English. The survey completion time was approximately 10 to 15 min. This study was approved by the Monash Health Human Research Ethics Committee and the local research governance office at each recruitment site.

### 2.2. Scales and Measures

The survey consisted of 42 items, including vaccine status, sociodemographics, cancer-related clinical details, and questions from three validated scales (Table S1):

1. Oxford COVID-19 Vaccine Hesitancy Scale (OHS), a 7-item scale measuring intent to receive a COVID-19 vaccine and vaccine hesitancy [32].
2. Oxford COVID-19 Vaccine Confidence and Complacency Scale (OCCS), a 14-item scale measuring attitudes around vaccine complacency and confidence. There are four identified factors: collective importance of a vaccine, belief that the vaccine will work, speed of vaccine development and side effects [32].
3. Disease Influenced Vaccine Acceptance Scale-Six (DIVAS-6), a 6-item scale evaluating the impact of cancer on participants’ attitudes towards COVID-19 vaccination [33]. This scale was developed by the study team and validated in people with serious underlying medical conditions including cancer, diabetes, and multiple sclerosis. It consists of two subscales: the Disease Complacency subscale, which assesses the degree to which a participant’s disease affected their perceived risk of COVID-19 infection, and the Vaccine Vulnerability subscale, which assesses how the participant’s cancer diagnosis and treatment affected their perceived benefits and risks of the vaccine.

Consistent with the instrument’s instructions, responses to items on all three scales were recorded on a 5-point Likert scale, with an additional “Don’t Know” option provided. Higher scores indicated a greater degree of negative attitude towards vaccination. Responses for three DIVAS-6 items (questions 26, 27, 28) were reversed to be consistent with this pattern (with higher scores indicating a more negative attitude).

### 2.3. Statistical Analysis

Summary and subscale scores for the OHS, OCCS and DIVAS-6 were calculated. “Don’t know” responses were not analyzed, consistent with the scales’ methodology [32,33,34,35]. Data cleaning was performed to remove unsubmitted incomplete, duplicate, and ineligible responses. Missing data were not imputed.

Sociodemographic, clinical data, summary and subscale scores were summarized using descriptive statistics. Variables with too few classifications were combined or removed on an analysis-by-analysis basis: this included combining no formal education level and primary education level and removing non-binary/other gender due to the small number of observations. Logistic regression was used to examine relationships between demographic and clinical factors and vaccination status (defined as having received one or more doses versus none). Linear regression was used to explore associations between demographic and clinical factors with scale summary and subscale scores. One-way ANCOVA was used to assess whether there were differences in scale summary and subscale scores between vaccination status. Time since study commencement was controlled in regression and ANCOVA to account for variation due to changing environmental circumstances. Hierarchical multivariable regression was conducted using demographic and disease-related variables that were significantly correlated at r > 0.1 with the outcome variable of interest (using Pearson’s and Spearman’s Rho). Cancer types were assessed as dichotomous variables compared to all other cancer types. Cancer types that showed a significant relationship with scale scores or vaccination status were assessed using hierarchical regression, controlling for significantly correlated (r > 0.1) demographic and disease-related variables. Alpha values of <0.05 were considered significant. Statistical analyses were conducted using SPSS Statistics Version 26.0 (IBM, Armonk, NY, USA).

## 3. Results

### 3.1. Patient Characteristics

Completed survey responses were received from 2691 people with solid organ cancers. Respondents had a median age of 62.5 years (*SD* = 11.8; range 19–95), 40.9% were male, 71.3% lived in metropolitan areas, and 90.3% spoke English as their dominant language (Table 1). The most common cancer types were breast (36.6%), genitourinary (18.6%) and gastrointestinal (18.3%); the cancer was localized in 59.2%, and 56.0% reported being currently on anti-cancer treatment.

### 3.2. COVID-19 Vaccine Uptake

**Figure 1:**
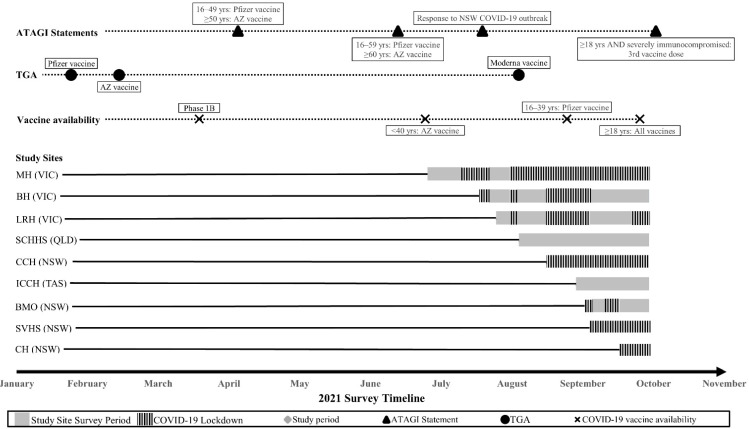
Timeline of study including survey period relative to state-wide strict lockdowns for COVID-19. Abbreviations: ATAGI, Australian Technical Advisory Group on Immunization; years, yrs; AZ, Astra-Zeneca; NSW, New South Wales; TGA, Therapeutic Goods Administration–COVID-19 Vaccine Provisional Registration; MH, Monash Health; VIC, Victoria; BH, Bendigo Health; LRH, Latrobe Regional Hospital; SCHHS; Sunshine Coast Hospital and Health Service; QLD; Queensland; CCH, Central Coast Hematology; ICCH, Icon Cancer Center Hobart; TAS, Tasmania; BMO, Border Medical Oncology; SVHS, St Vincent’s Hospital Sydney; CH, Campbelltown Hospital. People with solid organ tumors were eligible for COVID-19 vaccination from the commencement of the Australian Government Rollout Phase 1B. (please see Figure S1 for clearer figure)

**Figure 2:**
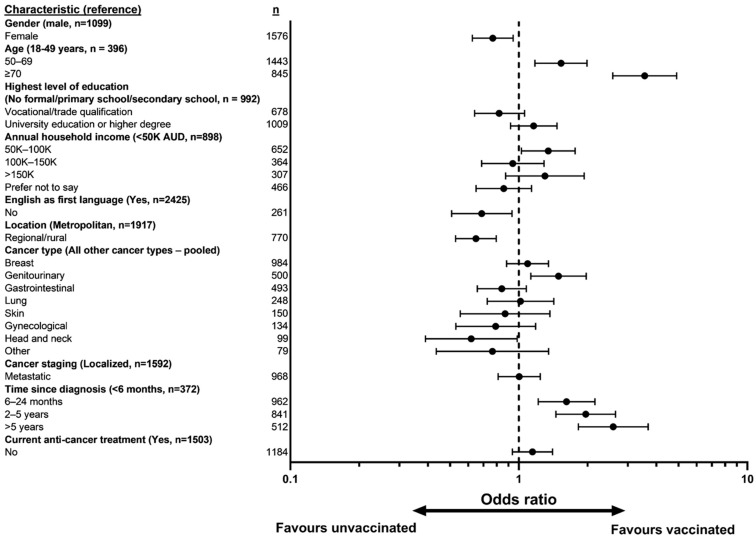
Forest plot of time-adjusted logistic regression model of factors associated with vaccine uptake. Abbreviations: AUD, Australian dollars; K, 1000 (please see Figure S2 for clearer figure).

The main features of the Australian vaccine rollout program and its relation to community transmission of COVID-19 and other public health measures are shown in Figure 1, giving context to the health and social environment at the time of the survey. The vaccination rate for the total study population was 79.7%. Female gender (OR 0.77, 95% CI 0.63, 0.95, *p* = 0.01), English as the non-dominant language (OR 0.69, 95% CI 0.51, 0.93, *p* = 0.02) and regional/rural location (OR 0.65, 95% CI 0.53, 0.80, *p* < 0.001) were associated with a significantly decreased likelihood of vaccine uptake (Figure 2 and Table S2). Conversely, older age (50–69 years (OR 1.53, 95% CI 1.18, 1.99, *p* = 0.002)) and annual household income AUD 50,000–AUD 100,000 (OR 1.35, 95% CI 1.03, 1.76, *p* = 0.03) were associated with a significantly higher likelihood of being vaccinated.

With regard to cancer characteristics (Figure 2 and Table S2), time-adjusted analysis showed higher vaccination rates amongst patients with a longer time since cancer diagnosis (6–24 months (OR 1.62, 95% CI 1.22, 2.15, *p* < 0.001), 2–5 years (OR 1.96, 95% CI 1.45, 2.65, *p* < 0.001), >5 years (OR 2.59, 95% CI 1.82, 3.68, *p* < 0.001)) and those for whom the primary site was in the genitourinary system (OR 1.49, 95% CI 1.13, 1.97, *p* = 0.005). People with head and neck cancer had a significantly lower likelihood of vaccine uptake (OR 0.62, 95% CI 0.39, 0.98, *p* = 0.04).

When significantly correlated (r > 0.1) demographic and cancer-related variables were entered into multivariable analysis, age, location, and time since cancer diagnosis remained significant predictors of vaccine status (Table S3). The relationship of being vaccinated with genitourinary cancer type was no longer significant after controlling for age, location, gender, time since cancer diagnosis and cancer stage (Table S4). The converse relationship with head and neck cancer was just shy of significance after controlling for age, location, gender, time since cancer diagnosis and current anti-cancer treatment (Table S5).

### 3.3. Oxford COVID-19 Vaccine Hesitancy Scale

Significantly higher Oxford COVID-19 Vaccine Hesitancy Scale summary scores, indicating greater vaccine hesitancy, were observed in unvaccinated respondents, (adjusted mean difference 7.38 (95% CI 6.93, 7.84, *p* < 0.001)).

When independently analyzed with linear regression, higher hesitancy scores were significantly associated with female gender, younger age and English as the non-dominant language (Table S6). When significantly correlated variables (r > 0.1) were entered together using multivariable analysis, age, university education and English as a non-dominant language remained significant (Table S7). Genitourinary cancer was associated with lower vaccine hesitancy scores (Table S6), although this did not remain significant after adjusting for relevant sociodemographic and clinical variables (Table S8).

### 3.4. Oxford COVID-19 Vaccine Confidence and Complacency Scale

Higher scores, indicating more prevalent concerns surrounding vaccine efficacy and side effects, were observed in unvaccinated participants. No significant demographic or cancer-related associations were seen (data not shown).

### 3.5. Disease Influenced Vaccine Acceptance Scale-6

**Figure 3:**
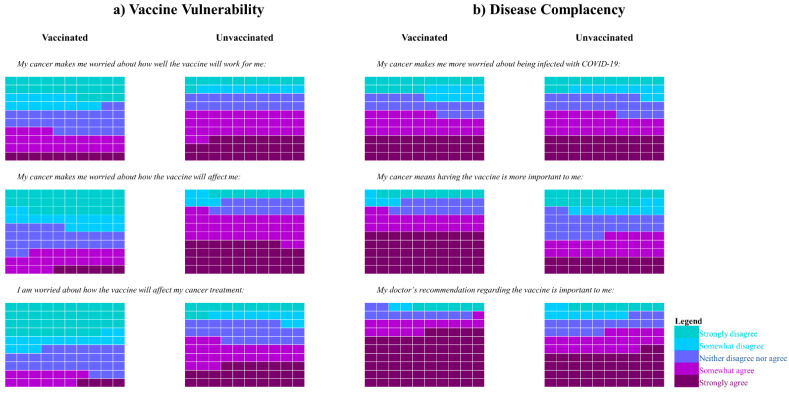
Response frequencies for the DIVAS-6 subscales: (**a**) Vaccine Vulnerability (**b**) Disease Complacency. Each box represents 1% of responses. Abbreviations: DIVAS-6, Disease Influenced Vaccine Acceptance Scale-6 (please see Figure S3 for clearer figure).

Significantly higher summary and subscale scores were observed in unvaccinated compared with vaccinated participants, who reported more concerns about vaccine efficacy (60.2% v. 34.2%), side effects (72.1% v. 28.9%) and interactions with anti-cancer treatment (53.4% v. 17.7%) (Figure 3a). Regarding the statement “cancer makes me more worried about being infected with COVID-19”, 56.8% agreed (comprising strongly agree or somewhat agree); this was independent of vaccination status (Figure 3b). The statement “my cancer means having the vaccine is more important to me” was agreed with by 67.2% overall (72.3% of vaccinated and 45.7% of unvaccinated participants). Vaccinated respondents were more likely to agree with the importance of a doctor’s recommendation regarding the vaccine (81.7% v. 66.0%).

**Figure 4:**
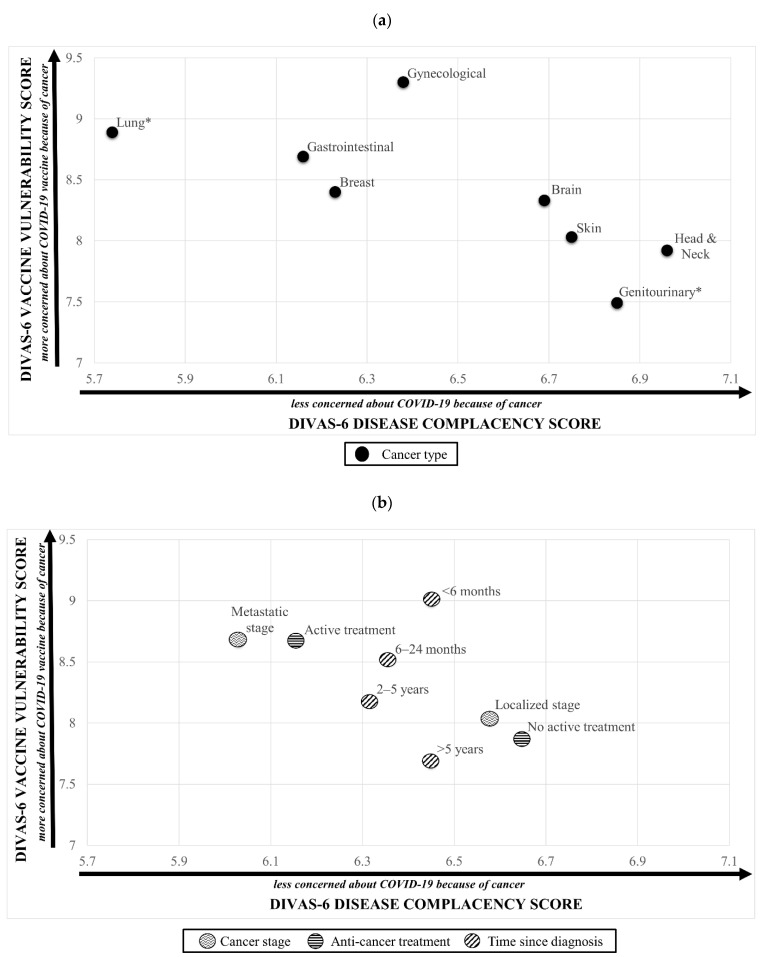
DIVAS-6 Disease Complacency against Vaccine Vulnerability scores by (**a**) cancer type (**b**) clinical characteristics. * indicates whether the cancer type, when compared with all other cancer types, was significant when individually regressed with both the DIVAS-6 Disease Complacency and DIVAS-6 Vaccine Vulnerability subscales’ scores. Note: Brain cancer was the most common cancer type reported within the ‘other’ cancers category (n = 46) and was included instead of the whole category. Abbreviations: DIVAS-6, Disease Influenced Vaccine Acceptance Scale-6. (please see Figure S4a,b for clearer figure)

Analysis of DIVAS-6 subscale scores across demographic and cancer characteristics identified differences in mean disease complacency and vaccine vulnerability scores (Figure 4, Tables S9 and S10). Participants with metastatic cancer, on active cancer treatment or with lung cancer reported significantly lower disease complacency coupled with significantly higher vaccine vulnerability scores, identifying participants who had greater concerns about contracting COVID-19 due to their cancer coupled with greater concerns about the effect of the vaccine on disease progression and treatments. Conversely, those with localized cancer, not on active treatment or with genitourinary cancer reported significantly higher disease complacency and significantly lower vaccine vulnerability scores, identifying participants who were less concerned about contracting COVID-19 due to their cancer coupled with having fewer vaccine-related concerns.

## 4. Discussion

This study comprehensively assessed COVID-19 vaccine hesitancy specifically in people with cancer using a suite of validated scales, including a disease-specific scale to assess the influence of having cancer on vaccine attitudes. Over half of our large and diverse sample expressed a perception of vulnerability regarding COVID-19 infection due to their underlying cancer, and this was irrespective of vaccination status. Concurrently, there were prominent concerns regarding the impact of cancer on vaccine efficacy, adverse effects, and interactions with anti-cancer treatments; this was particularly strong amongst unvaccinated participants.

Interestingly, the overall vaccination rate (79.7%) was not higher than the general Australian population at the time (80.5%), despite people with cancer having two extra months of access to free vaccines [36]. This finding potentially reflects the influence of the disease-specific factors faced by people with cancer, adding additional concerns to this complex decision-making process. Consistent with smaller studies, there were few differences in uptake and hesitancy between people with different cancer types and stages or treatment status [25,26,37,38]. In comparison to the original study conducted in a UK representative general population group, our respondents displayed a much lower mean score on the Oxford COVID-19 Vaccine Hesitancy Scale. This likely reflects the more medically vulnerable population in our study, the impact of having effective vaccines available and the reduction in vaccine hesitancy over the course of pandemic, a trend shown in a number of studies [32,39]. Despite this, significant concerns regarding vaccine efficacy and safety were evident as demonstrated by the present study, highlighting the ongoing need to address these concerns as the pandemic continues, especially given the recommendations for booster and additional vaccine doses in cancer populations [40,41,42].

The DIVAS-6 scale provides additional insight into how a cancer diagnosis impacts both the fear of COVID-19 disease and any hesitancy toward vaccination (and conversely, acceptance). It appears to be a useful addition to the two Oxford scales, which largely examine attitudes and beliefs independent of other factors affecting a person’s health. For example, increased hesitancy was observed in people diagnosed within six months and those with metastatic disease. This is consistent with previous studies which suggest that these groups would likely be susceptible to increased cancer-related anxiety due to increased uncertainty surrounding their conditions [43]. It follows that such patient groups, once identified, can be more specifically targeted by public information campaigns, as well as alerting individual clinicians that a discussion about how COVID-19 and vaccination may affect a particular patient may be required. Our findings suggest that patients from culturally and linguistically diverse backgrounds and those living in regional/rural areas would benefit from such targeted strategies.

Our survey reinforces previous studies showing that healthcare professionals are a trusted resource for patients with cancer [28,44,45]. In a Korean study of 1001 people with cancer, 61% indicated they were willing to accept a vaccine when available; this increased to 91% with a recommendation from their physician [38]. Furthermore, a physician-led educational intervention in the form of a webinar was demonstrated to increase patient understanding about vaccines and increase their intention to be vaccinated [46]. As such, clinicians should be harnessed as an important partner in vaccination campaigns, which is particularly relevant now it appears that ongoing boosters against SARS-CoV-2 will be required. Certainly, current behavioral science research has identified key communication strategies to address vaccine hesitancy, including recruiting trusted voices such as patient peers, advocates, and health providers to establish a positive social norm of vaccination in people with cancer [47].

Numerous professional bodies have produced guidelines and educational resources for patients and clinicians. At the start of the pandemic, these were based on expert opinion, but they now need to evolve to incorporate emerging data about vaccine efficacy and timing, tailored to different patient cohorts [48,49,50,51]. In addition to the challenge of keeping standard but tailored culturally appropriate information up to date, a focus on harmonized dissemination is required to ensure consistency of advice is provided by the many other practitioners encountered by people with cancer, as well as in public education campaigns [52]. We have previously documented the negative impact of receiving discordant information on people with cancer [29,53].

Insights provided by DIVAS-6 suggest it is a useful tool to facilitate individualized discussions between clinicians and patients with cancer. The two subscales elucidate the influences underlying a person’s attitude towards vaccination, whether having a cancer diagnosis raised perceived vulnerabilities to COVID-19 infection and therefore was a source of motivation for vaccination, or the contrary, where the main concerns were that vaccines could add risks to the person’s cancer or treatments. The six questions can be easily implemented in clinical environments and attitudes tracked over time, which is important given our data showing hesitancy reduced with longer times since diagnosis.

As with any survey, there are some limitations. The convenience sampling method means our results are a representation of those who chose to complete the survey. This bias, coupled with recall and misclassification bias inherent in survey research, should be considered with relation to broader generalizations drawn from these results. Our study population had a slightly greater proportion of females, likely related to the high proportion of participants with breast cancer. We did not evaluate broader cultural and political factors, which have been found to significantly affect attitudes towards vaccination such as social media usage, sociopolitical group association or adherence to COVID-19 vaccine conspiracy theories but rather focused on disease-related influences [54,55,56]. Additionally, the epidemiology of COVID-19 in Australia at the time of the study, with a comparably low prevalence and disease burden, may somewhat limit direct comparisons with other regions of the world; it is more likely much of the information will be valuable across many jurisdictions.

This was a large study of COVID-19 vaccine hesitancy in people with cancer, sampling across a diverse range of demographics and cancer-disease characteristics in Australia. The use of multiple validated tools, including disease-specific measures, has given a comprehensive picture of attitudes of this medically vulnerable group and can be used to target education to further raise vaccine compliance.

## 5. Conclusions

In summary, vaccine attitudes and hesitancy in people with cancer are substantially influenced by their underlying cancer diagnosis. Medical vulnerability to COVID-19 due to disease status or anti-cancer treatments can motivate and enable positive vaccine uptake; conversely, concerns regarding a potential negative impact of the vaccine on health, underlying cancer and treatments contributes to hesitancy. These complex concerns must be comprehensively addressed both in individual patients and in public health messaging to best protect this medically vulnerable population from the ongoing morbidity and mortality of the COVID-19 pandemic.

## Figures and Tables

**Table 1:** Participant characteristics.

	Total n = 2691 (%)	Vaccinated n = 2143 (79.7%)	Unvaccinated n = 546 (20.3%)
Male	1101 (40.9)	898 (81.7)	201 (18.3)
Female *	1578 (58.6)	1235 (78.3)	343 (21.7)
Age: mean (SD)	62.5 (11.8)	63.7 (11.5)	58.0 (12.0)
Age (years)			
18–49	396 (14.7)	270 (68.2)	126 (31.8)
50–69	1444 (53.8)	1113 (77.1)	331 (22.9)
≥70	847 (31.5)	757 (89.5)	89 (10.5)
Highest level of education **			
No formal/primary school	72 (2.7)	52 (72.2)	20 (27.8)
Secondary school	921 (34.3)	726 (78.8)	195 (21.2)
Vocational/Trade	679 (25.2)	526 (77.5)	153 (22.5)
University	1009 (37.5)	834 (82.7)	175 (17.3)
Annual household income (AUD)			
<50 K	898 (33.4)	694 (77.3)	204 (22.7)
50 K–100 K	653 (24.3)	543 (83.2)	110 (16.8)
100 K–150 K	364 (13.5)	286 (78.6)	78 (21.4)
>150 K	307 (11.4)	269 (87.6)	38 (12.4)
Prefer not to say	467 (17.4)	351 (75.2)	116 (24.8)
Aboriginal/Torres Strait Islander ***			
Yes	38 (1.4)	32 (84.2)	6 (15.8)
English as dominant language			
Yes	2429 (90.3)	1963 (80.9)	464 (19.1)
No	261 (9.7)	180 (69.0)	81 (31.0)
Location			
Metropolitan	1918 (71.3)	1594 (83.1)	324 (16.9)
Regional/rural	773 (28.7)	549 (71.2)	222 (22.8)
Cancer type			
Breast	986 (36.6)	807 (81.8)	179 (18.2)
Genitourinary	501 (18.6)	424 (84.8)	76 (15.2)
Gastrointestinal	493 (18.3)	375 (76.1)	118 (23.9)
Lung	248 (9.2)	194 (78.2)	54 (21.8)
Skin	151 (5.6)	123 (82.0)	27 (18.0)
Gynecological	134 (5.0)	91 (67.9)	43 (32.1)
Head and Neck	99 (3.7)	69 (69.1)	30 (30.3)
Other	79 (2.9)	60 (75.9)	19 (24.1)
Cancer stage			
Localized	1593 (59.2)	1291 (81.1)	301 (18.9)
Metastatic	971 (36.1)	770 (79.4)	200 (20.6)
Unsure/Other	127 (4.7)	82 (64.6)	45 (35.4)
Time since diagnosis			
<6 months	372 (13.8)	251 (67.5)	121 (32.5)
6–24 months	964 (35.8)	755 (78.4)	208 (21.6)
2–5 years	841 (31.3)	695 (82.6)	146 (17.4)
>5 years	514 (19.1)	442 (86.2)	71 (13.8)
Current anti-cancer treatment			
Yes	1506 (56.0)	1181 (78.5)	323 (21.5)
No	1185 (44.0)	962 (81.2)	223 (18.8)

Notes: exact cohort for vaccination status n = 2689. * does not include “non-binary/prefer not to say” n = 12 (0.4%), ** Other: 8 (0.3%); *** does not include “prefer not to say” n = 27 (1.0%). Abbreviations: AUD, Australian dollars; K, 1000.

## Data Availability

The data presented in this study are available on reasonable request from the corresponding author.

## Supplementary Materials

The following supporting information can be downloaded at: https://www.mdpi.com/article/10.3390/vaccines10091373/s1, List of CANVACCS investigators; Table S1: Online survey questions; Table S2: Logistic regression analysis predicting vaccinated status with sociodemographic and clinical characteristics; Table S3: Hierarchical multivariable logistic regression analysis of vaccinated status, with significantly correlated (r > 0.1) demographic and clinical variables; Table S4: Hierarchical multivariable logistic regression analysis of vaccinated status with genitourinary cancer type; Table S5: Hierarchical multivariable logistic regression analysis of vaccinated status with head and neck cancer; Table S6: Linear regression analysis predicting the Oxford COVID-19 Vaccine Hesitancy Scale summary score with sociodemographic and clinical characteristics; Table S7: Hierarchical multivariable linear regression analysis of the Oxford COVID-19 Vaccine Hesitancy Scale score, with significantly correlated (r > 0.1) demographic and disease-related variables; Table S8: Hierarchical multivariable linear regression analysis of the Oxford COVID-19 Vaccine Hesitancy Scale score with genitourinary cancer type; Table S9: Linear regression predicting the Disease Influenced Vaccine Acceptance Scale-Six (DIVAS-6) Disease Complacency subscale score with sociodemographic and clinical characteristics; Table S10: Linear regression predicting the Disease Influenced Vaccine Acceptance Scale-Six Vaccine Vulnerability subscale score with sociodemographic and clinical characteristics; Table S11: Hierarchical multivariable linear regression of the DIVAS-6 Disease Complacency subscale score with genitourinary cancer type; Table S12: Hierarchical multivariable linear regression analysis of the DIVAS-6 Vaccine Vulnerability subscale score with genitourinary cancer type. Figure S1. Timeline of study including survey period relative to state-wide strict lockdowns for COVID-19. Figure S2. Forest plot of time-adjusted logistic regression model of factors associated with vaccine uptake. Figure S3. Response frequencies for the DIVAS-6 subscales: (a) Vaccine Vulnerability (b) Disease Complacency. Figure S4a,b. DIVAS-6 Disease Complacency against Vaccine Vulnerability scores by (a) cancer type (b) clinical characteristics.

## Abbreviations

The following abbreviations are used in this manuscript:

95% CI	95% confidence interval
ATAGI	Australian Technical Advisory Group on Immunization
AUD	Australian Dollars
AZ	Astra-Zeneca
BH	Bendigo Health
BMO	Border Medical Oncology
CANVACCS	CANcer patients’ perspectives on coronavirus VACCination Survey
CCH	Central Coast Hematology
CH	Campbelltown Hospital
DIVAS-6	Disease Influenced Vaccine Acceptance Scale-6
ICCH	Icon Cancer Center Hobart
K	1000
LRH	Latrobe Regional Hospital
MH	Monash Health
NSW	New South Wales
OHS	Oxford COVID-19 Vaccine Hesitancy Scale
OCCS	Oxford COVID-19 Vaccine Confidence and Complacency Scale
OR	Odds ratio
QLD	Queensland
SCHHS	Sunshine Coast Hospital and Health Service
SVHS	St Vincent’s Hospital Sydney
r	correlation coefficient.
TAS	Tasmania
TGA	Therapeutic Goods Administration
v.	Versus
VIC	Victoria
yrs	years
