# Supplementary material for: COVID-19 Vaccine Hesitancy in Australian Patients with Solid Organ Cancers
Source: Vaccines (Basel). 2022 Aug 23;10(9):1373. doi: 10.3390/vaccines10091373 (PMC9503648; doi:10.3390/vaccines10091373)
Supplement: Supplementary file 1 [file vaccines-10-01373-s001.zip › Supplementary materials 22-08-22 clean.pdf]

Supplementary Materials

These supplementary materials have been provided by the authors to give readers additional information about their work.

Supplement to: **COVID-19 vaccine hesitancy in Australian patients with solid organ cancers.**

List of CANcer patients' perspectives on coronavirus VACCination Survey (CANVACCS) Investigators:

Monash Health, Victoria: Dr. Veronica Lopez Aedo, Dr. Elizabeth Ahern, Dr. Muhammad Alamgeer, Dr. Nathan Bain, Dr. Amy Body, A/Prof. Peter Briggs, Dr. Daphne Day, Dr. Sophia Frentzas, Dr. Lisa Grech, A/Prof. Marion Harris, Dr. Gwo-Yaw Ho, Dr. Caroline Lum, Dr. Vi Luong, Dr. Amelia McCartney, Dr. Cameron McLaren, Dr. Mike Nguyen, Prof. Stephen Opat, A/Prof. David Pook, Prof. Eva Segelov, A/Prof. Andrew Strickland, Dr. Avraham Travers, Dr. Kate Webber, Dr. Michelle White, Dr. Walid Zwieky.
Bendigo Health, Victoria: Dr. Sam Harris.
Latrobe Regional Hospital, Victoria: Dr. Hieu Chau.
Sunshine Coast Hospital and Health Service, Queensland: A/Prof. Bryan Chan.
Icon Cancer Centre Hobart, Tasmania: A/Prof. Louise Nott.
Central Coast Hematology, New South Wales: Dr. Richard Blennerhassett, Dr. Cecily Forsyth, Ms. Jacqueline Jagger.
St Vincent's Hospital Sydney, New South Wales: A/Prof. Nada Hamad.
Campbelltown Hospital, New South Wales: Dr. Annette Tognela.
Border Medical Oncology Research Unit, New South Wales: A/Prof. Craig Underhill.

Table S1. Online survey questions

**Screening items**	
Are you 18 years or older?	o Yes<br>o No (Terminate if No)
Have you received a cancer diagnosis?	o Yes<br>o No (Terminate if No)
Are you a [participating site] patient?	o Yes<br>o No (Terminate if No)
**Vaccination status**	
Have you already received a COVID-19 vaccine?	o Yes, 1 dose only<br>o Yes, 2 doses<br>o No
**Oxford COVID-19 Vaccine Hesitancy Scale**<br>Instructions: We would like to know your feelings and thoughts about the COVID-19 vaccine. Note: If you have already been vaccinated against COVID-19, please complete these questions in relation to a future COVID-19 vaccine dose/booster.	
Would you take a COVID-19 vaccine if offered?	o Definitely/have taken<br>o Probably<br>o I may or may not<br>o Probably not<br>o Definitely not<br>o Don't know
When a COVID-19 vaccine is available:	o I will want to get it as soon as possible<br>o I will take it when offered<br>o I'm not sure what I will do<br>o I will put off (delay) getting it<br>o I will refuse to get it<br>o Don't know
I would describe my attitude towards receiving a COVID-19 vaccine as:	o Very keen<br>o Pretty positive<br>o Neutral<br>o Quite uneasy<br>o Against it<br>o Don't know
If a COVD-19 vaccine was available in my local area, I would:	o Get it as soon as possible<br>o Get it when I have time<br>o Delay getting it<br>o Avoid getting it for as long as possible<br>o Never get it<br>o Don't know
If my family or friends were thinking of getting a COVID-19 vaccination, I would:	o Strongly encourage them<br>o Encourage them<br>o Not say anything to them about it<br>o Ask them to delay getting the vaccination<br>o Suggest that they do not get the vaccination<br>o Don't know
I would describe myself as:	o Eager to get a COVID-19 vaccine<br>o Willing to get the COVID-19 vaccine<br>o Not bothered about getting the COVID-19 vaccine<br>o Unwilling to get the COVID-19 vaccine<br>o Anti-vaccination for COVID-19<br>o Don't know
Taking a COVID-19 vaccination is:	o Really important<br>o Important<br>o Neither important nor unimportant<br>o Unimportant<br>o Really unimportant<br>o Don’t know
**Oxford COVID-19 Vaccine Confidence and Complacency Scale**	
Do you think you will be infected with COVID-19 over the next 12 months?	o Definitely<br>o Probably<br>o Possibly

	o Probably not<br>o Definitely not<br>o Don't know
I think the COVID-19 vaccine is likely to:	o Work for almost everyone<br>o Work for most people<br>o I am unsure how many people it will work for<br>o Not work for most people<br>o Not work for anyone<br>o Don't know
I think the COVID-19 vaccine is likely to:	o Definitely work for me<br>o Probably work for me<br>o May or may not work for me<br>o Probably not work for me<br>o Definitely not work for me<br>o Don't know
I think if I get the COVID-19 vaccine it will be:	o Really helpful for the community around me<br>o Helpful for the community around me<br>o Neither helpful nor unhelpful for the community around me<br>o Unhelpful for the community around me<br>o Really unhelpful for the community around me<br>o Don't know
I think if individuals like me get the COVID-19 vaccine it will:	o Save a large number of lives<br>o Save some lives<br>o Have no impact<br>o Lead to more deaths<br>o Lead to a large number of deaths<br>o Don't know
I think the speed of developing and testing the vaccine means it will be:	o Really good<br>o Good<br>o Will not affect how good or bad it is<br>o Bad<br>o Really bad<br>o Don't know
I think the speed of developing and testing the vaccine means it will be:	o Really safe<br>o Safe<br>o It will not affect how safe it is<br>o Unsafe<br>o Really unsafe<br>o Don't know
I think if many people do not get the vaccine this:	o Will be dangerous<br>o May be dangerous<br>o Will have no consequences at all<br>o May be good<br>o Will be good<br>o Don't know
I expect that receiving the vaccine will be:	o Hardly noticeable<br>o A little unpleasant<br>o Moderately unpleasant<br>o Painful<br>o Extremely painful<br>o Don't know
I think the side-effects for people of getting the COVID-19 vaccine will be:	o None<br>o Mild<br>o Moderate<br>o Significant<br>o Life-threatening<br>o Don't know
I think the COVID-19 vaccine will:	o Greatly strengthen my immune system<br>o Strengthen my immune system<br>o It will neither strengthen nor weaken my immune system<br>o Weaken my immune system<br>o Greatly weaken my immune system<br>o Don't know

I think taking the COVID-19 vaccine:	o Will give me complete freedom to get on with life just as before<br>o Will give me greater freedom<br>o Will have no effect on my freedom<br>o Will restrict my freedom<br>o Will completely restrict my freedom to get on with life<br>o Don't know
I think getting the vaccine is a sign of:	o Great personal strength<br>o Personal strength<br>o Not a sign of personal strength or weakness<br>o Personal weakness<br>o Great personal weakness<br>o Don't know
Taking a new COVID-19 vaccine will make me feel like a guinea pig:	o Do not agree<br>o Agree a little<br>o Agree moderately<br>o Agree a lot<br>o Completely agree<br>o Don't know
**Disease Influenced Vaccine Acceptance Scale-Six**<br><br>Instructions: We would like to know about how your cancer diagnosis may be related to your feelings and thoughts about the COVID-19 vaccine. For each of the following statements, please tap/click the one choice that best represents how strongly you agree or disagree with it. There are 6 choices to choose from for each statement.	
My history of cancer makes me more worried about being infected with COVID-19:	o Strongly agree<br>o Somewhat agree<br>o Neither disagree nor agree<br>o Somewhat disagree<br>o Strongly disagree<br>o Don't know
My history of cancer means having the vaccine is more important to me:	o Strongly agree<br>o Somewhat agree<br>o Neither disagree nor agree<br>o Somewhat disagree<br>o Strongly disagree<br>o Don't know
My doctor's recommendation regarding the vaccine is important to me:	o Strongly agree<br>o Somewhat agree<br>o Neither disagree nor agree<br>o Somewhat disagree<br>o Strongly disagree<br>o Don't know
My history of cancer makes me worried about how well the vaccine will work for me:	o Strongly disagree<br>o Somewhat disagree<br>o Neither disagree nor agree<br>o Somewhat agree<br>o Strongly agree<br>o Don't know
My history of cancer makes me worried about how the vaccine will affect me:	o Strongly disagree<br>o Somewhat disagree<br>o Neither disagree nor agree<br>o Somewhat agree<br>o Strongly agree<br>o Don't know
I am worried about how the vaccine will affect my cancer treatment:	o Strongly disagree<br>o Somewhat disagree<br>o Neither disagree nor agree<br>o Somewhat agree<br>o Strongly agree<br>o Don't know
**Clinical**	
What type of cancer do you have? (i.e. where your cancer started and not where it has spread to).	o Breast<br>o Lung (including mesothelioma)<br>o Genitourinary (i.e. prostate, kidney, testicular or bladder)<br>o Skin (including melanoma)

	o Gastrointestinal (i.e. stomach, esophagus, bile duct, gallbladder, pancreas, colon, rectum, or anus)<br>o Gynecological (i.e. ovarian, cervical, uterine or vulvar/vaginal)<br>o Head and neck (i.e. mouth, throat, sinus or nose)<br>o Brain<br>o Blood (i.e. leukemia, myeloma and lymphoma)<br>o Other (please specify):___________________________
When was your cancer diagnosed?	o Less than 6 months ago<br>o 6 to 24 months ago<br>o 2 to 5 years ago<br>o More than 5 years ago
As you understand it, is your cancer in just the one area where it started (localized) or has it spread to other places in the body (metastatic)?	o Localized<br>o Metastatic<br>o Don't know<br>o Other (please type any comments:__________________)
Are you currently on cancer treatment?	o Yes<br>o No
How long ago was your last treatment? (e.g. chemotherapy, immunotherapy, hormonal treatment, targeted therapy, radiotherapy and/or clinical trial).	o Currently on treatment<br>o Less than 1 year ago<br>o 1 to 5 years ago<br>o More than 5 years ago
Socio-demographics	
What is your gender?	o Male<br>o Female<br>o Non-binary /third gender<br>o Prefer not to say
What is your age?	_________________
What is your highest educational level (completed)?	o No formal education<br>o Primary education<br>o Secondary education<br>o Vocational/trade qualification<br>o University education or higher degree<br>o Other (please specify): _____________________
What is your annual household income (including everyone who lives in your home)?	o Less than $50,000<br>o $50,001 to $100,000<br>o $100,001 to $150,000<br>o More than $150,000<br>o Prefer not to say
Do you identify as Aboriginal and/or Torres Strait Islander?	o Yes<br>o No<br>o Prefer not to say
Is English your first language?	o Yes<br>o No
Please include any comments about your feelings and thoughts about your cancer and COVID-19 vaccination that you would like to share. If you have no comments to include, please type 'Nil'.	[Text entry]:<br>____

Table S2. Logistic regression analysis predicting vaccinated status with sociodemographic and clinical characteristics.

Category (reference)	n	B (SE)	Odds ratio (95% CI)	p-value
**Gender (Male)**	2675			
Female		-0.26 (0.11)	0.77 (0.63 – 0.95)	0.01
**Age (18–49 years)**	2684			
50–69		0.43 (0.13)	1.53 (1.18 – 1.99)	0.002
≥70		1.27 (0.16)	3.56 (2.58 – 4.91)	<0.001
**Highest level of education (No formal /primary school/secondary school)**	2679			
Vocational/Trade		-0.20 (0.13)	0.82 (0.64 – 1.06)	0.13
University		0.15 (0.12)	1.16 (0.92 – 1.47)	0.21
**Annual household income (AUD<50K)**	2687			
50K–100K		0.30 (0.14)	1.35 (1.03 – 1.76)	0.03
100K–150K		-0.06 (0.16)	0.94 (0.69 – 1.29)	0.70
>150K		0.26 (0.20)	1.30 (0.88 – 1.93)	0.19
Prefer not to say		-0.15 (0.14)	0.86 (0.65 – 1.14)	0.29
**English as dominant language (Yes)**	2686			
No		-0.37 (0.16)	0.69 (0.51 – 0.93)	0.02
**Location (Metropolitan)**	2687			
Regional/rural		-0.43 (0.10)	0.65 (0.53 – 0.80)	<0.001
**Cancer Type (All other cancer types – pooled)**	2687			
Breast		0.09 (0.11)	1.09 (0.88 – 1.35)	0.42
Genitourinary		0.40 (0.14)	1.49 (1.13 – 1.97)	0.005
Gastrointestinal		-0.17 (0.13)	0.84 (0.66 – 1.08)	0.17
Lung		0.02 (0.17)	1.02 (0.73 – 1.42)	0.92
Skin		-0.14 (0.23)	0.87 (0.55 – 1.37)	0.55
Gynecological		-0.23 (0.21)	0.79 (0.53 – 1.18)	0.26
Head and Neck		-0.48 (0.24)	0.62 (0.39 – 0.98)	0.04
Other		-0.27 (0.29)	0.77 (0.44 – 1.35)	0.36
**Cancer Stage (Localized)**	2560			
Metastatic		0.004 (0.11)	1.00 (0.81 – 1.24)	0.97
**Time since diagnosis (<6 months)**	2687			
6–24 months		0.48 (0.15)	1.62 (1.22 – 2.15)	0.001
2–5 years		0.67 (0.15)	1.96 (1.45 – 2.65)	<0.001
>5 years		0.95 (0.18)	2.59 (1.82 – 3.68)	<0.001
**Current anti-cancer treatment (Yes)**	2687			
No		0.14 (0.10)	1.15 (0.94 – 1.40)	0.18

Notes: Regression analyses was controlled for time since study commencement. These variables were excluded due to <50 responses; Aboriginal and/or Torres Strait Islander status: non-binary/other gender, and “other” educational level. Cancer stage (don’t know/other) were excluded for comparisons between localized and metastatic. Abbreviations: B, unstandardized coefficient; SE, standard error; 95% CI, 95% confidence interval; AUD, Australian Dollars; K, 1000.

Table S3. Hierarchical multivariable logistic regression analysis of vaccinated status, with significantly correlated (r>0.1) demographic and clinical variables (cancer type not included) (n = 2684).

	**B**	**SE**	**Wald**	**df**	**p-value**	**OR**	**95% CI**	
							**Lower**	**Upper**
**Time since study commencement**	0.03	0.002	205.10	1	<0.001	1.03	1.03	1.04
**Age (years)**								
18–49 (reference)	-	-	-	-	-	-	-	-
50–69	0.44	0.14	10.39	1	0.001	1.55	1.19	2.02
≥70	1.27	0.17	58.07	1	<0.001	3.56	2.57	4.94
**Location**								
Metropolitan (reference)	-	-	-	-	-	-	-	-
Regional/rural	-0.47	0.11	19.54	1	<0.001	0.62	0.51	0.77
**Time since diagnosis**								
<6 months (reference)	-	-	-	-	-	-	-	-
6–24 months	0.50	0.15	11.29	1	0.001	1.65	1.23	2.20
2–5 years	0.69	0.16	19.67	1	<0.001	2.00	1.47	2.72
>5 years	0.83	0.18	20.43	1	<0.001	2.29	1.60	3.28
Constant	-1.03	0.19	30.61	1	<0.001	0.36		

Notes: Variables entered at each step: Step 1, time since study commencement; Step 2, age and location; Step 3, time since diagnosis. Variables significantly correlated (r>0.1) with vaccinated status: age, location, time since diagnosis. Abbreviations: B, unstandardized coefficient; SE, standard error; df, degrees of freedom; OR, odds ratio; 95% CI, 95% confidence interval.

Table S4. Hierarchical multivariable logistic regression analysis of vaccinated status with genitourinary cancer type, compared with all other cancer types, controlling for significantly correlated (r>0.1) demographic and disease-related variables with either vaccinated status or genitourinary cancer type (when compared with all other cancer types) (n = 2545).

	**B**	**SE**	**Wald**	**df**	**p-value**	**OR**	**95% CI**	
							**Lower**	**Upper**
**Time since study commencement**	0.03	0.002	185.25	1	<0.001	1.03	1.03	1.04
**Age (years)**								
18–49 (reference)	-	-	-	-	-	-	-	-
50–69	0.46	0.14	10.37	1	0.001	1.58	1.20	2.08
≥70	1.27	0.18	50.77	1	<0.001	3.57	2.51	5.06
**Location**								
Metropolitan (reference)	-	-	-	-	-	-	-	-
Regional/rural	-0.51	0.11	20.95	1	<0.001	0.60	0.48	0.75
**Gender**								
Male (reference)	-	-	-	-	-	-	-	-
Female	-0.06	0.13	0.23	1	0.63	0.94	0.73	1.21
**Genitourinary cancer type**	0.06	0.18	0.11	1	0.74	1.06	0.75	1.50
**Time since diagnosis**								
<6 months (reference)	-	-	-	-	-	-	-	-
6–24 months	0.45	0.18	8.46	1	0.004	1.57	1.16	2.13
2–5 years	0.63	0.16	14.84	1	<0.001	1.87	1.36	2.58
>5 years	0.76	0.19	15.10	1	<0.001	2.13	1.45	3.11
**Cancer stage**								
Localized (reference)	-	-	-	-	-	-	-	-
Metastatic	-0.15	0.12	1.59	1	0.21	0.87	0.69	1.08
Constant	-0.84	0.22	14.08	1	<0.001	0.43		

Notes: Variables entered into the model at each step: Step 1, time since study commencement; Step 2, age, location, gender; Step 3, genitourinary cancer type, time since diagnosis, cancer stage. Variables significantly correlated (r>0.1) with vaccinated status: age, location, time since diagnosis. Variables significantly correlated (r>0.1) with genitourinary cancer type: age, gender, time since diagnosis, cancer stage. Variable categories excluded from analysis: Non-binary/prefer not to say (gender); don't know/not applicable/other (cancer stage). Abbreviations: B, unstandardized coefficient; SE, standard error; df, degrees of freedom; OR, odds ratio; 95% CI, 95% confidence interval.

Table S5. Hierarchical multivariable logistic regression analysis of vaccinated status with head and neck cancer, compared with all other cancer types, controlling for significantly correlated (r>0.1) demographic and disease-related variables with either vaccinated status or head and neck cancer type (when compared with all other cancer types) (n = 2545).

	B	SE	Wald	df	p-value	OR	OR 95% CI	
							Lower	Upper
**Time since study commencement**	0.03	0.002	203.88	1	<0.001	1.03	1.03	1.04
**Age (years)**								
18–49 (reference)	-	-	-	-	-	-	-	-
50–69	0.43	0.14	9.53	1	0.002	1.53	1.17	2.00
≥70	1.23	0.17	51.77	1	<0.001	3.44	2.45	4.81
**Location**								
Metropolitan (reference)	-	-	-	-	-	-	-	-
Regional/rural	-0.48	0.11	19.60	1	<0.001	0.62	0.50	0.77
**Gender**								
Male (reference)	-	-	-	-	-	-	-	-
Female	-0.09	0.11	0.65	1	0.42	0.91	0.73	1.14
**Head and neck cancer type**	-0.47	0.25	3.56	1	0.059	0.63	0.39	1.02
**Time since diagnosis**								
<6 months (reference)	-	-	-	-	-	-	-	-
6–24 months	0.45	0.15	8.76	1	0.003	1.56	1.16	2.10
2–5 years	0.64	0.16	16.15	1	<0.001	1.90	1.39	2.60
>5 years	0.81	0.19	19.10	1	<0.001	2.25	1.56	3.24
**Current anti-cancer treatment**								
Yes (reference)	-	-	-	-	-	-	-	-
No	0.11	0.11	1.07	1	0.30	1.12	0.90	1.39
Constant	-0.95	0.21	20.93	1	<0.001	0.39		

Notes: Variables entered into the model at each step: 1, time since study commencement; Step 2, age, location, gender; Step 3, head and neck cancer, time since diagnosis, current anti-cancer treatment. Variables significantly correlated with vaccinated status with r>0.1 were: age, location, time since diagnosis. Variables significantly correlated with head and neck cancer type with r>0.1 were: gender, current anti-cancer treatment. Variable categories excluded from analysis: Non-binary/prefer not to say (gender). Abbreviations: B, unstandardized coefficient; SE, standard error; df, degrees of freedom; OR, odds ratio; 95% CI, 95% confidence interval.

Table S6. Linear regression analysis predicting the Oxford COVID-19 Vaccine Hesitancy Scale summary score with sociodemographic and clinical characteristics.

		Step 1	Step 2				
**Category (reference)**	**n**	**Adj. R^2**	**Adj. R^2**	**Δ Adj. R^2**	**B (SE)**	**t**	**p-value**
**Gender (Male)**	2497	0.023	0.025	0.002			
Female					0.42 (0.20)	2.15	0.03
**Age (18–49 years)**	2505	0.024	0.041	0.017			
50–69					-1.35 (0.28)	-4.76	<0.001
≥70					-2.08 (0.30)	-6.87	<0.001
**Highest level of education (No formal /primary school/secondary school)**	2504	0.024	0.027	0.003			
Vocational/Trade					0.40 (0.25)	1.60	0.11
University					-0.42 (0.22)	-1.86	0.06
**Annual household income (AUD<50K)**	2508	0.024	0.027	0.003			
50–100K					-0.42 (0.26)	-1.64	0.10
100K–150K					-0.16 (0.31)	-0.52	0.60
>150K					-0.65 (0.33)	-1.96	0.05
Prefer not to say					0.45 (0.29)	1.55	0.12
**English as dominant language (Yes)**	2507	0.024	0.029	0.005			
No					1.22 (0.33)	3.69	<0.001
**Location (Metropolitan)**	2508	0.024	0.025	0.001			
Regional/rural					0.48 (0.22)	2.12	0.034
**Cancer Type (All other cancer types – pooled)**	2508						
Breast		0.024	0.024	0.00	0.32 (0.20)	1.58	0.12
Genitourinary		0.024	0.025	0.001	-0.58 (0.25)	-2.33	0.02
Gastrointestinal		0.024	0.024	0.00	-0.19 (0.25)	-0.74	0.46
Lung		0.024	0.024	0.00	-0.45 (0.34)	-1.33	0.18
Skin		0.024	0.024	0.00	0.70 (0.42)	1.67	0.10
Gynecological		0.024	0.023	-0.001	0.26 (0.46)	0.56	0.58
Head and Neck		0.024	0.025	0.001	0.98 (0.51)	1.91	0.06
Other		0.024	0.023	-0.001	-0.14 (0.57)	-0.25	0.81
**Cancer Stage (Localized)**	2404	0.024	0.024	0.00			
Metastatic					-0.13 (0.20)	-0.64	0.52
**Time since diagnosis (<6 months)**	2508	0.024	0.024	0.00			
6–24 months					-0.23 (0.31)	-0.74	0.46
2–5 years					-0.22 (0.31)	-0.71	0.48
>5 years					-0.64 (0.34)	-1.86	0.062
**Current anti-cancer treatment (Yes)**	2508	0.024	0.023	-0.001			
No					-0.029 (0.20)	-0.15	0.88

Notes: Step 1, time since study commencement is the only predictor variable entered into the model; Step 2, the clinic-demographic is the predictor variable entered into the model. These variables were excluded due to <50 responses; Aboriginal and/or Torres Strait Islander status: non-binary/other gender, "other"' educational level. Cancer stage (don't know/other) were excluded for comparisons between localized and metastatic. Analyses were not undertaken for Aboriginal and/or Torres Strait Islander status, due to limited observations. Abbreviations: Adj. R^2, Adjusted R^2; B(SE), unstandardized coefficient (standard error); t, t-statistic; AUD, Australian Dollars; K, 1000.

Table S7. Hierarchical multivariable linear regression analysis of the Oxford COVID-19 Vaccine Hesitancy Scale score, with significantly correlated (r>0.1) demographic and disease-related variables (n = 2500).

**Step and predictor variable**	**B**	**SE**	**t**	**p-value**	**sr**	**Adj. R^2**	**ΔAdj. R^2**
Step 1						0.024	0.024
Constant	11.30	0.22	52.46	<0.001			
**Time since study commencement**	-0.03	0.004	-7.87	<0.001	-0.16		
Step 2						0.052	0.028
Constant	12.72	0.35	36.76	<0.001			
**Time since study commencement**	-0.03	0.004	-6.83	<0.001	-0.14		
**Age (years)**							
18–49 (reference)	-	-	-	-	-		
50–69	-1.43	0.28	-5.03	<0.001	-0.10		
≥70	-2.22	0.31	-7.24	<0.001	-0.14		
**Highest level of education**							
No formal/primary school/secondary school (reference)	-	-	-	-	-		
Vocational/trade qualification	0.17	0.25	0.69	0.49	0.01		
University	-0.81	0.23	-3.61	<0.001	-0.07		
**English as dominant language**							
Yes (reference)	-	-	-	-	-		
No	1.24	0.33	3.78	<0.001	0.08		

Notes: Variables entered into the model at each step: Step 1, time since study commencement; Step 2, age, highest level of education, English as dominant language. Variables significantly correlated (r>0.1) with the Oxford COVID-19 Vaccine Hesitancy Scale score: age, highest level of education, English as dominant language. Variable categories excluded from analysis: Other (Highest level of education). Abbreviations: B, unstandardized coefficient; SE, standard error; t, t-statistic; sr, semipartial correlation coefficient; Adj. R^2, Adjusted R^2.

Table S8. Hierarchical multivariable linear regression analysis of the Oxford COVID-19 Vaccine Hesitancy Scale score with genitourinary cancer type, compared with all cancer types, controlling for significantly correlated (r>0.1) demographic and disease-related variables with either the Oxford COVID-19 Vaccine Hesitancy Scale score or genitourinary cancer type (when compared with all other cancer types) (n = 2385).

Step and predictor variable	B	SE	t	p-value	sr	Adj. R^2	Δ Adj. R^2
Step 1						0.024	0.024
Constant	11.24	0.22	51.16	<0.001			
**Time since study commencement**	-0.03	0.004	-7.71	<0.001	-0.16		
Step 2						0.050	0.026
Constant	12.55	0.39	32.61	<0.001			
**Time since study commencement**	-0.03	0.004	-6.84	<0.001	-0.14		
**Age (years)**							
18–49 (reference)	-	-	-	-	-		
50–69	-1.42	0.29	-4.92	<0.001	-0.10		
≥70	-2.12	0.32	-6.62	<0.001	-0.13		
**Gender**							
Male (reference)	-	-	-	-	-		
Female	0.15	0.20	0.73	0.47	0.015		
**Highest level of education**							
No formal/primary school/secondary school (reference)	-	-	-	-	-		
Vocational/trade qualification	0.25	0.26	0.96	0.34	0.02		
University	-0.74	0.23	-3.23	0.001	-0.07		
**English as dominant language**							
Yes (reference)	-	-	-	-	-		
No	1.01	0.34	2.93	0.003	0.06		
Step 3						0.048	-0.002
Constant	12.79	0.46	27.78	<0.001			
**Time since study commencement**	-0.03	0.004	-6.72	<0.001	-0.14		
**Age (years)**							
18–49 (reference)	-	-	-	-	-		
50–69	-1.40	0.29	-4.84	<0.001	-0.10		
≥70	-2.09	0.32	-6.45	<0.001	-0.13		
**Gender**							
Male (reference)	-	-	-	-	-		
Female	0.10	0.24	0.43	0.67	0.01		
**Highest level of education**							
No formal/primary school/secondary school (reference)	-	-	-	-	-		
Vocational/trade qualification	0.25	0.26	0.97	0.33	0.02		
University	-0.73	0.23	-3.16	0.002	-0.07		
**English as dominant language**							
Yes (reference)	-	-	-	-	-		
No	1.00	0.35	2.90	0.004	0.06		
**Genitourinary cancer type**	-0.11	0.30	-0.35	0.73	-0.007		
**Time since diagnosis**							
<6 months (reference)	-	-	-	-	-		
6–24 months	-0.29	0.31	-0.94	0.35	-0.02		

2–5 years	-0.24	0.32	-0.76	0.45	-0.02		
>5 years	-0.33	0.35	-0.92	0.36	-0.02		
**Cancer stage**							
Localized (reference)	-	-	-	-	-		
Metastatic	0.02	0.21	0.08	0.94	0.002		

Notes: Variables entered into the model at each step: Step 1, time since study commencement; Step 2, age, gender, highest level of education, English as dominant language; Step 3, genitourinary cancer type, time since diagnosis, cancer stage. Variables significantly correlated (r>0.1) with the Oxford COVID-19 Vaccine Hesitancy Scale score: age, highest level of education, English as dominant language. Variables significantly correlated (r>0.1) with genitourinary cancer type: age, gender, time since diagnosis, cancer stage. Variable categories excluded from analysis: Non-binary/prefer not to say (gender); other (highest educational level); don't know/not applicable/other (cancer stage). Abbreviations: B, unstandardized coefficient; SE, standard error; t, t-statistic; sr, semipartial correlation coefficient; Adj. R^2, Adjusted R^2.

Table S9. Linear regression predicting the Disease Influenced Vaccine Acceptance Scale-Six (DIVAS-6) Disease Complacency subscale score with sociodemographic and clinical characteristics.

		Step 1	Step 2				
**Category (reference)**	**n**	**Adj. R^2**	**Adj. R^2**	**Δ Adj. R^2**	**B (SE)**	**t**	**p-value**
**Gender (Male)**	2524	0.009	0.012	0.003			
Female					-0.34 (0.12)	-2.76	0.01
**Age (18–49 years)**	2532	0.009	0.01	0.001			
50–69					0.21 (0.18)	1.20	0.23
≥70					0.40 (0.19)	2.08	0.04
**Highest level of education (No formal /primary school/secondary school)**	2529	0.009	0.008	-0.001			
Vocational/Trade					-0.04 (0.16)	-0.28	0.78
University					-0.11 (0.14)	-0.78	0.44
**Annual household income (AUD<50K)**	2535	0.009	0.009	0.00			
50K–100K					-0.09 (0.16)	-0.57	0.57
100K–150K					-0.22 (0.19)	-1.13	0.26
>150K					0.02 (0.21)	0.11	0.91
Prefer not to say					0.18 (0.18)	1.01	0.31
**English as dominant language (Yes)**	2534	0.009	0.009	0.00			
No					-0.05 (0.21)	-0.25	0.81
**Location (Metropolitan)**	2535	0.009	0.009	0.00			
Regional/rural					-0.01 (0.14)	-0.05	0.96
**Cancer Type (All other cancer types – pooled)**	2535						
Breast		0.009	0.01	0.001	-0.18 (0.13)	-1.45	0.15
Genitourinary		0.009	0.015	0.006	0.60 (0.15)	3.88	<0.001
Gastrointestinal		0.009	0.01	0.001	-0.30 (0.16)	-1.92	0.06
Lung		0.009	0.014	0.005	-0.74 (0.21)	-3.54	<0.001
Skin		0.009	0.01	0.001	0.46 (0.27)	1.72	0.09
Gynecological		0.009	0.009	0.00	-0.15 (0.28)	-0.55	0.58
Head and Neck		0.009	0.01	0.001	0.53 (0.32)	1.67	0.10
Other		0.009	0.011	0.002	0.75 (0.35)	2.11	0.04
**Cancer Stage (Localized)**	2426	0.009	0.017	0.008			
Metastatic					-0.59 (0.13)	-4.66	<0.001
**Time since diagnosis (<6 months)**	2535	0.009	0.008	-0.001			
6–24 months					-0.05 (0.19)	-0.25	0.80
2–5 years					-0.05 (0.20)	-0.24	0.81
>5 years					0.07 (0.21)	0.34	0.73
**Current anti-cancer treatment (Yes)**	2535	0.009	0.016	0.007			
No					0.51 (0.12)	4.19	<0.001

Notes: Step 1 = time since study commencement is the only predictor variable entered into the model; Step 2 = the clinic-demographic is the predictor variable entered into the model. These variables were excluded due to <50 responses; Aboriginal and/or Torres Strait Islander status: non-binary/other gender, "other"' educational level. Cancer stage (don't know/other) were excluded for comparisons between localized and metastatic. Abbreviations: Adj. R^2, Adjusted R^2; B(SE), unstandardized coefficient (standard error); t, t-statistic; AUD, Australian Dollars; K, 1000.

Table S10. Linear regression predicting the Disease Influenced Vaccine Acceptance Scale-Six (DIVAS-6) Vaccine Vulnerability subscale score with sociodemographic and clinical characteristics.

		Step 1	Step 2				
**Category (reference)**	**n**	**Adj. R^2**	**Adj. R^2**	**Δ Adj. R^2**	**B (SE)**	**t**	**p-value**
**Gender (Male)**	2316	0.005	0.015	0.01			
Female					0.74 (0.15)	5.02	<0.001
**Age (18–49 years)**	2325	0.005	0.03	0.025			
50–69					-0.69 (0.21)	-3.31	0.001
≥70					-1.65 (0.23)	-7.28	<0.001
**Highest level of education (No formal /primary school/secondary school)**	2324	0.005	0.015	0.01			
Vocational/Trade					0.05 (0.19)	0.28	0.78
University					-0.73 (0.17)	-4.32	<0.001
**Annual household income (AUD<50K)**	2328	0.005	0.017	0.012			
50-100K					-0.38 (0.20)	-1.96	0.05
100K-150K					0.07 (0.23)	0.32	0.75
>150K					-0.74 (0.25)	-3.00	0.003
Prefer not to say					0.67 (0.22)	3.03	0.002
**English as dominant language (Yes)**	2327	0.005	0.017	0.012			
No					1.36 (0.25)	5.38	<0.001
**Location (Metropolitan)**	2328	0.005	0.005	0.00			
Regional/rural					0.14 (0.17)	0.79	0.43
**Cancer Type (All other cancer types – pooled)**	2328						
Breast		0.005	0.005	0.00	0.15 (0.15)	0.98	0.33
Genitourinary		0.005	0.017	0.012	-1.00 (0.19)	-5.36	<0.001
Gastrointestinal		0.005	0.007	0.002	0.41 (0.19)	2.17	0.03
Lung		0.005	0.007	0.002	0.58 (0.26)	2.25	0.03
Skin		0.005	0.005	0.00	-0.27 (0.32)	-0.83	0.40
Gynecological		0.005	0.007	0.002	0.87 (0.36)	2.43	0.02
Head and Neck		0.005	0.006	0.001	-0.50 (0.40)	-1.26	0.21
Other		0.005	0.005	0.00	0.16 (0.44)	0.37	0.71
**Cancer Stage (Localized)**	2237	0.004	0.011	0.007			
Metastatic					0.62 (0.15)	4.05	<0.001
**Time since cancer diagnosis (<6 months)**		0.005	0.016	0.011			
6–24 months					-0.43 (0.23)	-1.89	0.06
2–5 years					-0.75 (0.24)	-3.15	0.002
>5 years					-1.25 (0.26)	-4.84	<0.001
**Current anti-cancer treatment (Yes)**	2328	0.005	0.017	0.012			
No					-0.79 (0.15)	-5.33	<0.001

Notes: Step 1 = time since study commencement is the only predictor variable entered into the model; Step 2 = the clinic-demographic is the predictor variable entered into the model. These variables were excluded due to <50 responses; Aboriginal and/or Torres Strait Islander status: non-binary/other gender, "other"' educational level. Cancer stage (don't know/other) were excluded for comparisons between localized and metastatic. Abbreviations: Adj. R^2, Adjusted R^2; B(SE), unstandardized coefficient (standard error); t, t-statistic; AUD, Australian Dollars; K, 1000.

Table S11. Hierarchical multivariable linear regression of the DIVAS-6 Disease Complacency subscale score with genitourinary cancer type, compared with all other cancer types, controlling for significantly correlated (r>0.1) demographic and disease-related variables with either DIVAS-6 Disease Complacency subscale score or genitourinary cancer type (when compared with all other cancer types) (n = 2412).

**Step and predictor variable**	**B**	**SE**	**t**	**p-value**	**sr**	**Adj. R^2**	**Δ Adj. R^2**
Step 1						0.009	0.009
Constant	6.95	0.14	50.29	<0.001			
**Time since study commencement**	-0.01	0.002	-4.68	<0.001	-0.10		
Step 2						0.011	0.002
Constant	6.96	0.22	31.62	<0.001			
**Time since study commencement**	-0.01	0.002	-4.74	<0.001	-0.10		
**Age (years)**							
18–49 (reference)	-	-	-	-	-		
50–69	0.16	0.18	0.89	0.38	0.02		
≥70	0.29	0.20	1.45	0.15	0.03		
**Gender**							
Male (reference)	-	-	-	-	-		
Female	-0.31	0.13	-2.34	0.02	-0.05		
Step 3						0.026	0.015
Constant	7.09	0.28	25.73	<0.001			
**Time since study commencement**	-0.01	0.002	-5.17	<0.001	-0.10		
**Age (years)**							
18–49 (reference)	-	-	-	-	-		
50–69	0.20	0.18	1.10	0.27	0.02		
≥70	0.28	0.20	1.38	0.17	0.03		
**Gender**							
Male (reference)	-	-	-	-	-		
Female	-0.19	0.15	-1.22	0.22	-0.02		
**Genitourinary cancer type**	0.61	0.19	3.15	0.002	0.06		
**Time since diagnosis**							
<6 months (reference)	-	-	-	-	-		
6–24 months	-0.02	0.20	-0.10	0.92	-0.002		
2–5 years	0.005	0.20	0.02	0.98	0.00		
>5 years	0.06	0.23	0.28	0.78	0.006		
**Cancer stage**							
Localized (reference)	-	-	-	-	-		
Metastatic	-0.74	0.13	-5.66	<0.001	-0.11		

Notes: Variables entered into the model at each step: Step 1, time since study commencement; Step 2, age, gender; Step 3, genitourinary cancer type, time since diagnosis, cancer stage. Variables significantly correlated (r>0.1) with the DIVAS-6 Disease Complacency subscale score: Nil. Variables significantly correlated (r>0.1) with genitourinary cancer type: age, gender, time since diagnosis, cancer stage. Variable categories excluded from analysis: Non-binary/prefer not to say (gender); don't know/not applicable/other (cancer stage). Abbreviations: B, unstandardized coefficient; SE, standard error; t, t-statistic; sr, semipartial correlation coefficient; Adj. R^2, Adjusted R^2.

Table S12. Hierarchical multivariable linear regression analysis of the DIVAS-6 Vaccine Vulnerability subscale score with genitourinary cancer type, compared with all other cancer types, controlling for significantly correlated (r>0.1) demographic and disease-related variables with either DIVAS-6 Vaccine Vulnerability subscale score or genitourinary cancer type (when compared with all other cancer types) (n = 2221).

	**B**	**SE**	**t**	**p-value**	**sr**	**Adj. R^2**	**Δ Adj. R^2**
Step 1						0.004	0.004
Constant	8.74	0.17	52.66	<0.001			
**Time since study commencement**	-0.01	0.003	-3.13	0.002	-0.07		
Step 2						0.041	0.037
Constant	8.93	0.26	34.16	<0.001			
**Time since study commencement**	-0.006	0.003	-2.23	0.03	-0.05		
**Age (years)**							
18–49 (reference)	-	-	-	-	-		
50–69	-0.55	0.21	-2.58	0.01	-0.05		
≥70	-1.40	0.24	-5.88	<0.001	-0.12		
**Gender**							
Male (reference)	-	-	-	-	-		
Female	0.48	0.16	3.08	0.002	0.06		
**English as dominant language**							
Yes (reference)	-	-	-	-	-		
No	1.17	0.26	4.46	<0.001	0.09		
Step 3						0.068	0.037
Constant	9.40	0.33	28.52	<0.001			
**Time since study commencement**	-0.004	0.003	-1.55	0.12	-0.03		
**Age (years)**							
18–49 (reference)	-	-	-	-	-		
50–69	-0.55	0.21	-2.64	0.008	-0.05		
≥70	-1.34	0.24	-5.69	<0.001	-0.12		
**Gender**							
Male (reference)							
Female	0.33	0.18	1.84	0.07	0.04		
**English as dominant language**							
Yes (reference)	-	-	-	-	-		
No	1.07	0.26	4.13	<0.001	0.09		
**Genitourinary cancer type**	-0.55	0.23	-2.43	0.02	-0.05		
**Time since diagnosis**							
<6 months (reference)	-	-	-	-	-		
6–24 months	-0.28	0.23	-1.20	0.23	-0.03		
2–5 years	-0.57	0.24	-2.37	0.02	-0.05		
>5 years	-0.96	0.27	-3.61	<0.001	-0.07		
**Cancer stage**							
Localized (reference)	-	-	-	-	-		
Metastatic	0.80	0.16	4.95	<0.001	0.10		
**Current anti-cancer treatment**							
Yes (reference)	-	-	-	-	-		
No	-0.56	0.16	-3.56	<0.001	-0.07		

Notes: Variables entered into the model at each step: Step 1, time since study commencement; Step 2, age, gender, English as dominant language; Step 3, genitourinary cancer type, time since diagnosis, cancer stage, current anti-cancer treatment. Variables significantly correlated (r>0.1) with DIVAS-6 Vaccine Vulnerability subscale: age, gender, English as dominant language, time since diagnosis, cancer stage, current anti-cancer treatment. Variables significantly correlated (r>0.1) with genitourinary cancer type: age, gender, time since diagnosis, cancer stage. Variable categories excluded from analysis: Non-binary/prefer not to say (gender); don't know/not applicable/other (cancer stage). Abbreviations: B, unstandardized coefficient; SE, standard error; t, t-statistic; sr, semipartial correlation coefficient; Adj. R^2, Adjusted R^2.
